# Macrophage migration inhibitory factor: a mediator of matrix metalloproteinase-2 production in rheumatoid arthritis

**DOI:** 10.1186/ar2021

**Published:** 2006-07-26

**Authors:** Angela Pakozdi, Mohammad A Amin, Christian S Haas, Rita J Martinez, G Kenneth Haines, Lanie L Santos, Eric F Morand, John R David, Alisa E Koch

**Affiliations:** 1 University of Michigan Medical School, 109 Zina Pitcher Place, Ann Arbor, MI 48109, USA; 2 Northwestern University Feinberg School of Medicine, 251 E. Huron Street, Chicago, IL 60611, USA; 3 Monash University Department of Medicine, Monash Medical Centre, Locked Back No 29, Clayton VIC 3168, Australia; 4 Harvard School of Public Health, Boston, 665 Huntington Avenue, Boston, MA 02115, USA; 5 VA Medical Service, Department of Veterans Affairs, 2215 Fuller Road, Ann Arbor, MI 48105, USA

## Abstract

Rheumatoid arthritis (RA) is a chronic inflammatory disease characterized by destruction of bone and cartilage, which is mediated, in part, by synovial fibroblasts. Matrix metalloproteinases (MMPs) are a large family of proteolytic enzymes responsible for matrix degradation. Macrophage migration inhibitory factor (MIF) is a cytokine that induces the production of a large number of proinflammatory molecules and has an important role in the pathogenesis of RA by promoting inflammation and angiogenesis.

In the present study, we determined the role of MIF in RA synovial fibroblast MMP production and the underlying signaling mechanisms. We found that MIF induces RA synovial fibroblast MMP-2 expression in a time-dependent and concentration-dependent manner. To elucidate the role of MIF in MMP-2 production, we produced zymosan-induced arthritis (ZIA) in *MIF *gene-deficient and wild-type mice. We found that MMP-2 protein levels were significantly decreased in *MIF *gene-deficient compared with wild-type mice joint homogenates. The expression of MMP-2 in ZIA was evaluated by immunohistochemistry (IHC). IHC revealed that MMP-2 is highly expressed in wild-type compared with *MIF *gene-deficient mice ZIA joints. Interestingly, synovial lining cells, endothelial cells, and sublining nonlymphoid mononuclear cells expressed MMP-2 in the ZIA synovium. Consistent with these results, in methylated BSA (mBSA) antigen-induced arthritis (AIA), a model of RA, enhanced MMP-2 expression was also observed in wild-type compared with *MIF *gene-deficient mice joints. To elucidate the signaling mechanisms in MIF-induced MMP-2 upregulation, RA synovial fibroblasts were stimulated with MIF in the presence of signaling inhibitors. We found that MIF-induced RA synovial fibroblast MMP-2 upregulation required the protein kinase C (PKC), c-jun N-terminal kinase (JNK), and Src signaling pathways. We studied the expression of MMP-2 in the presence of PKC isoform-specific inhibitors and found that the PKCδ inhibitor rottlerin inhibits MIF-induced RA synovial fibroblast MMP-2 production. Consistent with these results, MIF induced phosphorylation of JNK, PKCδ, and c-jun. These results indicate a potential novel role for MIF in tissue destruction in RA.

## Introduction

Rheumatoid arthritis (RA) is a chronic inflammatory disease characterized by destruction of bone and cartilage, which is mediated, in part, by synovial fibroblasts. Matrix metalloproteinases (MMPs) are a large family of proteolytic enzymes responsible for degradation of extracellular matrix components and are thought to have a crucial role in RA joint destruction [1]. MMPs are classified into five subgroups according to their structural domains and substrate specificity:

1. Collagenases, such as interstitial collagenase (MMP-1), neutrophil collagenase (MMP-8), and collagenase-3 (MMP-13).

2. Gelatinases, including gelatinase A (MMP-2) and gelatinase B (MMP-9).

3. Stromelysins, such as stromelysin-1 (MMP-3) and stromelysin-2 (MMP-10).

4. Membrane-type MMPs (MT-MMPs), including MT1-MMP, MT2-MMP, MT3-MMP, MT4-MMP, MT5-MMP, and MT6-MMP.

5. Other MMPs, such as matrilysin, stromelysin-3, metalloelastase, enamelysin, and MMP-19.

Despite distinct classification, the role of each individual MMP in a specific process, such as RA, is not clear yet. However, MMPs are thought to participate in extracellular matrix degradation in several pathologic conditions, including bone remodeling, atherosclerosis, apoptosis, angiogenesis, tumor invasion, and RA [2-10].

Most MMPs are secreted as latent proenzymes and their activation requires proteolytic degradation of the propeptide domain. This activation occurs extracellularly and is often mediated by activated MMPs [11]. A number of different stimuli are known to promote MMP-2 activation through MT1-MMP, such as proteinase-3, neutrophil elastase, cathepsin G, and thrombin [12,13]. The present study focuses on MMP-2, which might contribute to the invasive characteristic features of the RA synovial fibroblast. MMP-2 degrades gelatin, collagen (types I, II, III, IV, V, VII, and X), fibronectin, elastin, and laminin [14]. MMP-2 is secreted by fibroblasts, keratinocytes, epithelial cells, monocytes, and osteoblasts [15].

Previous data suggest that MMP-2 has an important role in RA. RA patients with radiographic erosions have significantly higher levels of active MMP-2 in their synovial tissues than patients without erosions, suggesting that MMP-2 has a crucial role in articular destruction [16]. In addition, MMP-2 has been previously linked to invasion of RA synovial fibroblasts [17,18] and implicated in angiogenesis [7,19]. Elevated MMP levels (MMP-1, MMP-2, MMP-3, MMP-7, MMP-8, MMP-9, and MMP-13) are detected in RA compared with osteoarthritis synovial fluid [20]. In the RA synovium, MMP-2 is expressed in the lining and sublining layers, in addition to the synovial membrane–cartilage interface [21,22].

Macrophage migration inhibitory factor (MIF) was originally identified as a protein derived from T lymphocytes [23,24]. MIF is a proinflammatory cytokine produced by macrophages in response to inflammatory stimuli such as TNF-α or IFN-γ [25]. MIF induces the production of a large number of proinflammatory molecules, such as TNF-α, IFN-γ, IL-1β, IL-6, IL-8, nitric oxide, and cyclo-oxygenase 2 (COX2) [25-28]. Recently, we and others showed MIF to be an important cytokine in angiogenesis [29,30] and the pathogenesis of RA [31]. Several independent studies described MIF enhancing angiogenesis and having a role in tumor neovascularization [32,33]. In type II collagen-induced arthritis, a murine model of RA, treatment with neutralizing anti-MIF antibodies delays the onset, and decreases the frequency, of arthritis [31]. Moreover, *MIF *gene-deficient mice exhibit significantly less synovial inflammation than wild-type mice after arthritis induction with type II collagen [34].

The purpose of the present study was to investigate the role and mechanism of action of MIF in RA synovial fibroblast MMP-2 production, which might lead to tissue degradation in RA, and describe significant signaling events leading to MIF-induced MMP-2 upregulation.

## Materials and methods

### Antibodies and reagents

Recombinant human MIF, recombinant human TNF-α (tumor necrosis factor-alpha, ED_50_, 0.02–0.05 ng/ml), recombinant human MMP-2, and recombinant human MMP-9 were purchased from R&D Systems (Minneapolis, MN, USA). The following specific inhibitors were obtained from Calbiochem (La Jolla, CA, USA): phosphatidylinositol 3-kinase (PI3K) inhibitor, LY294002; mitogen-activated protein kinase extracellular-signal-regulated kinase (MAPK/ERK (MEK)) inhibitor, PD98059; Src inhibitor, PP2; janus kinase (Jak) inhibitor, AG490; nuclear factor-κB (NF-κB) inhibitor, pyrrolidine dithiocarbamate (PDTC); p38 mitogen-activated protein kinase (MAPK) inhibitor, SB203580; c-jun N-terminal kinase (JNK) inhibitor II; protein kinase C (PKC) inhibitor, Ro-31-8425; specific PKCαβ inhibitor, Gö 6976; PKCδ inhibitor, rottlerin; and signal transducer and activator of transcription (STAT) 3 inhibitor peptide. The G-protein inhibitor pertussis toxin was purchased from Sigma (St Louis, MO, USA). Inhibitors were dissolved in distilled water or dimethyl sulfoxide (DMSO) according to the manufacturer's instructions. Rabbit antihuman phospho-specific antibodies, directed against phosphorylated forms of PKC (pan; Thr514), PKCα_I_β_II _(Thr638/641), PKCδ (Tyr311), PKCδ (Thr505), stress-activated protein kinase SAPK/JNK (Thr183/Tyr185), and c-Jun (Ser63) antibodies were obtained from Cell Signaling Technology (Beverly, MA, USA). Rabbit antihuman phospho-PKCε (Ser729) was purchased from Upstate (Lake Placid, NY, USA). Mouse antihuman MMP-2 was purchased from R&D Systems, rabbit antimouse MMP-2 was obtained from Novus Biologicals (Littleton, CO, USA). Goat antirabbit alkaline phosphatase-conjugated antibody and rabbit antihuman actin antibody were obtained from Sigma. Alexa Fluor-488 donkey antimouse immunoglobulin (Ig) G, Alexa Fluor-555 donkey antirabbit IgG, and 4',6-diamidino-2-phenylindole dihydrochloride (DAPI) were purchased from Molecular Probes Inc. (Eugene, OR, USA). RPMI 1640, Dulbecco's PBS and fetal bovine serum (FBS) were purchased from Invitrogen (Carlsbad, CA, USA).

### Cell culture

Human RA synovial fibroblasts were isolated from synovial tissue obtained from RA patients who had undergone synovectomy or total-joint-replacement surgery. The protocol for patient consent and the use of human tissues was approved by the Institutional Review Board at both the University of Michigan (Ann Arbor, MI, USA) and the University of Michigan Health Systems (Ann Arbor, MI, USA). All tissue was obtained with patient consent. Fresh synovial tissues were minced and digested in a solution of dispase, collagenase, and DNase. The cells were cultured in RPMI 1640 supplemented with 10% FBS in 175-mm tissue-culture flasks at 37°C in a humidified atmosphere with 5% carbon dioxide (CO_2_). On reaching confluence, the cells were passaged by brief trypsinization, as described by Koch *et al*. [35]. Cells were used at passage 5–9, at which time they were a homogeneous, 85–95% confluent population of fibroblasts. The medium was switched to serum-free 12–14 hours before the experiments. The concentrations of cytokines and signaling inhibitors used in the experiments were derived from those used by our laboratory and others. RA synovial fibroblast cell viability with inhibitors, using trypan blue exclusion, was >95%.

### Animals

*MIF *gene-deficient mice were generated as described previously by Bozza *et al*. [36]; SV129/J wild-type mice served as controls. Mice were maintained and bred in a specific pathogen-free facility at the University of Michigan according to the guidelines for animal research. Animal experiments were in concordance with federal law and were performed after approval by the University Committee in Use and Care of Animals.

### Induction of arthritis

Zymosan-induced arthritis (ZIA) was induced by intra-articular injection of zymosan (*Saccharomyces cerevisiae*), as follows: zymosan was prepared by dissolving 30 mg of zymosan in 1 ml of sterile PBS. The solution was boiled twice and sonicated. Mice were anesthetized with pentobarbital (60 mg/kg body weight intraperitoneally) and injected with zymosan (10 μl) into each knee joint [37]. After 24 hours, mice were euthanized and ZIA knees were harvested: one knee was homogenized in PBS containing protease inhibitors (Protease Inhibitor Cocktail, Boehringer Mannheim, Mannheim, Germany), using a Polytron homogenizer (Brinkmann, Westbury, NY, USA), while the other knee was stored in frozen tissue matrix (Tissue-Tek O.C.T. Compound, Sakura Finetek, Torrance, CA, USA).

Antigen-induced arthritis (AIA) was induced in *MIF *gene-deficient and wild-type mice as described previously by Yang *et al*. [38]. The AIA model involves both cellular and humoral immune responses and shows histologic similarities to human RA. Briefly, mice were immunized at day 0 with 200 μg of methylated BSA (mBSA; Sigma), which was emulsified in 0.2 ml of Freund's complete adjuvant and injected subcutaneously into the flank skin. At day 7, mice received 100 μg mBSA in 0.1 ml Freund's complete adjuvant by intradermal injection into the base of the tail. At day 21, arthritis was induced by intra-articular injection of mBSA (30 μg in 10 μl of sterile PBS) into the knee. On day 28, mice were euthanized and AIA knees were harvested and homogenized in PBS containing protease inhibitors.

### Quantitation of MMPs by enzyme immunoassay (ELISA)

The concentrations of MMP-1, MMP-2, MMP-3 and MMP-13 in cell culture supernatants and MMP-2 in mouse knee homogenates were measured using Quantikine immunoassay kits (R&D Systems) according to the manufacturer's instructions. To maintain equal protein loading, protein concentrations of ZIA knee homogenates were determined using a bicinchoninic acid (BCA) assay (Pierce, Rockford, IL, USA). ELISAs detect both the proforms and active forms of MMP-2 and MMP-3, and solely the proforms of MMP-1 and MMP-13.

### Cell number determination

We used a CyQuant cell-enumeration kit (Invitrogen) to monitor equal cell numbers in the presence or absence of stimuli. RA synovial fibroblasts (× 10^4^) were plated into 96-well plates in RPMI containing 10% FBS. The night before the assay, the medium was replaced with serum-free RPMI. Cells were incubated in the presence or absence of MIF (50 nM) for 24 hours and the cell number was evaluated with CyQuant. Fluorescence background in CyQuant-treated wells without cells was subtracted from all values.

### Cell lysis and western blotting

After treatment with MIF, cells were lysed with lysis buffer (175 μl; Cell Signaling Technology) containing protease inhibitors. The concentration of protein in each extract was determined using a BCA protein assay, with BSA as the standard. SDS-PAGE was performed with cell lysates after equal protein loading, according to the method of Laemmli [39], and proteins were transferred onto a nitrocellulose membrane using a semidry transblotting apparatus (Bio-Rad, Hercules, CA, USA). Nitrocellulose membranes were blocked with 5% nonfat milk in Tris-buffered saline Tween (TBST) buffer (20 mM Tris, 137 mM NaCl, and 0.1% Tween 20 at pH 7.6) for 60 minutes at room temperature. Blots were incubated with phospho-specific antibodies at 1:1000 in TBST buffer containing 5% nonfat milk overnight at 4°C. Blots were washed with TBST buffer (three times) for 10 minutes (on each occasion) and incubated with antirabbit horseradish peroxidase-conjugated antibodies at room temperature. After washing three times for 10 minutes (on each occasion) with TBST buffer, blots were incubated with enhanced chemiluminescence (ECL) reagents (Amersham Biosciences, Piscataway, NJ, USA) according to the manufacturer's instructions. The immunoblots were stripped and re-probed with rabbit anti-β-actin to verify equal loading.

### Gelatinase assay

Gelatinase activity of RA synovial fibroblast culture media was measured using EnzChek gelatinase assay kits (Invitrogen). Cell culture supernatants were incubated with fluorescein-conjugated gelatin (100 μg/ml) for 3 hours, and fluorescence was measured using a Synergie HT microplate reader at 495 nm (Biotek, Winooski, VT, USA).

### Gelatin zymography

The MMP-2 enzyme secreted by RA synovial fibroblasts was analyzed on gelatin-containing gels, as previously described by Stetler-Stevenson *et al*. [40]. Additionally, gelatin degradation was visualized in AIA joint homogenates after equalizing the protein concentration using a BCA protein assay. To the standard acrylamide mixture, B-type gelatin (Sigma) was added to make a final concentration of 1 mg/ml. Samples were mixed with an equal volume of 2 × sample buffer (which consisted of 10% SDS, 10% glycerol, 0.5 M Tris-HCl, and 0.1% bromophenol blue at pH 6.8) and then added to 7.5% SDS-polyacrylamide gels (SDS-PAGE) for 2 hours. Following electrophoresis, gels were renatured in 2.5% Triton X-100 for 1 hour at room temperature. The gels were then incubated at 37°C overnight in developing buffer (which consisted of 50 mM Tris-HCl, 0.2 M NaCl, and 5 mM CaCl_2_). Gels were stained for 3 hours with Coomassie Brilliant Blue R-250 (Bio-Rad) and then destained with destaining solution (which consisted of 7.5% acetic acid and 5% methanol). Gelatinase activities were visualized as white bands on the blue background of the gels. Molecular-weight marker (Sigmamarker, Sigma) and recombinant human matrix metalloproteinase-2 (rhMMP-2) were used as controls. Photographs of the zymograms were taken with a Nikon Coolpix 4500 (Nikon, Melville, NY, USA) digital camera.

### Immunohistology

Frozen tissue (wild-type and *MIF *gene-deficient mouse ZIA joints) were cut (approximately 7 μm) and stained using alkaline phosphatase and fast red substrate for visualization. Slides were fixed in cold acetone for 10 minutes and then rehydrated with tris-buffered saline (TBS) solution for 2 minutes. Tissues were blocked with 20% FBS and 5% goat serum (in TBS) for 15 minutes at room temperature and then incubated with rabbit antimouse MMP-2 (diluted 1:200, in blocking buffer) or purified nonspecific rabbit IgG for 1 hour at room temperature. The tissue was washed three times in TBS, and a 1:100 dilution of goat antirabbit alkaline phosphatase-conjugated antibody (in blocking buffer) was added to the tissue sections before incubation for an additional 1 hour. After washing three times in TBS, slides were developed with Naphtol AS-MX Phosphate and Fast Red TR Salt (for 20 minutes at room temperature; Pierce), rinsed in tap water, counterstained with Gill's hematoxylin, and dipped in saturated lithium carbonate solution for bluing. Staining was evaluated under blinded conditions and graded by a pathologist. Slides were examined for cellular immunoreactivity and cell types were distinguished according to their characteristic morphology. The percentage of cells expressing MMP-2 was analyzed in the synovial lining cells (fibroblast-like and macrophage-like synoviocytes), subsynovial nonlymphoid mononuclear cells (monocytes, macrophages, and mast cells), and on endothelial cells.

### Immunofluorescence staining

RA synovial fibroblasts were plated at 3,000 cells/well (in eight-well chamber slides) in RPMI with 5% FBS overnight. The next day, the media was changed to serum-free RPMI. After serum starvation overnight, cells were stimulated with MIF (50 nM) for 20 minutes. The media was aspirated and the cells were washed with PBS and fixed with ice-cold methanol for 30 minutes. Blocking was performed by adding 5% FBS in PBS for 1 hour at room temperature. Phospho-specific primary antibodies for JNK and c-jun, or anti-MMP-2 antibody (diluted 1:50 in blocking buffer), were added overnight at 4°C. Cells were washed with PBS three times for 5 minutes on each occasion. Alexa Fluor-conjugated secondary antibodies, diluted 1:200 in blocking buffer, were added for 1 hour at room temperature. Cells were washed with PBS three times for 5 minutes on each occasion, and then DAPI nuclear stain was added for 5 minutes at a 1:2000 dilution in PBS. Slides were dehydrated, mounted, and covered with coverslips. Immunofluorescence staining was detected using an Olympus BX51 Fluorescence Microscope System with DP Manager imaging software (Olympus America, Melville, NY, USA).

### Statistical analysis

Data were analyzed using the student's *t*-test, assuming equal variance. *P *values <0.05 were considered statistically significant. Data are represented as the mean ± standard error of the mean (SEM).

## Results

### MIF induces the production of MMP-2 in RA synovial fibroblasts

**Figure 1:**
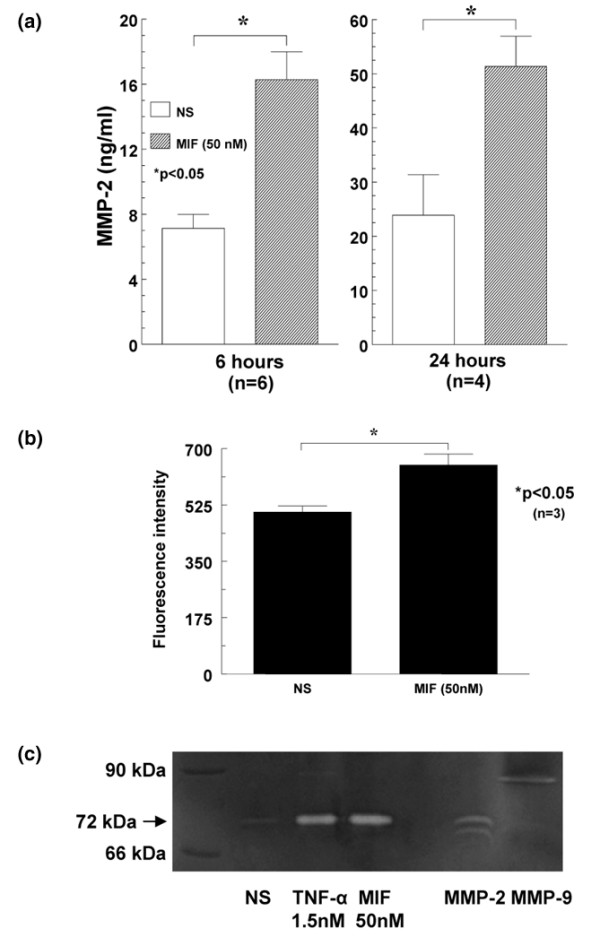
MIF induces MMP-2 production by rheumatoid arthritis (RA) synovial fibroblasts. **(a) **RA synovial fibroblast MMP-2 production was measured in supernatants by ELISA after 6-hour (left) and 24-hour (right) incubation, with or without MIF (50 nM). MIF-stimulated RA synovial fibroblasts produced twice as much MMP-2 as controls. The mean concentration of MMP-2 ± standard error of the mean (SEM) is represented; *P < 0.05 (*n *= number of donors). **(b) **Gelatinase activity of RA synovial fibroblasts was measured using fluorescein-conjugated gelatin as substrate. Supernatants from 24-hour MIF-stimulated (50 nM) RA synovial fibroblasts had elevated gelatinase activity compared with nonstimulated fibroblasts. The mean fluorescence intensity ± SEM is represented; *P < 0.05 (*n *= number of donors). **(c) **MMP-2 production by RA synovial fibroblasts was measured by gelatin zymography. Molecular-weight marker and standard recombinant human MMP-2 and MMP-9 served as controls. Results are a single representative experiment of four independent experiments using RA synovial fibroblasts from four donors. MIF, macrophage migration inhibitory factor; MMP, matrix metalloproteinase; NS, nonstimulated; TNF-α, tumor necrosis factor-α.

RA synovial fibroblasts were stimulated with MIF (50 nM) for different time periods (6 hours, 24 hours, and 48 hours). Pro-MMP-1, total MMP-2 (proform plus active form), total MMP-3, and pro-MMP-13 concentrations in cell culture supernatants were measured by ELISA. Under the conditions described, MIF stimulation showed no effect on secretion of MMP-1, MMP-3, and MMP-13 proteins, because these proteins were not detected in 48-hour MIF-stimulated RA synovial fibroblast culture media by ELISA, whereas control experiments with TNF-α (1.5 nM) increased the concentration of MMP-1, MMP-3, and MMP-13 in the supernatants (data not shown). By contrast, MIF-stimulated RA synovial fibroblasts produced significantly higher amounts of MMP-2 protein compared with nonstimulated controls (Figure 1a). This effect was seen after 6 hours' stimulation (nonstimulated, 7.13 ± 0.86 ng/ml of MMP-2 and MIF-stimulated, 16.28 ± 1.71 ng/ml of MMP-2; *P *< 0.05) and also after 24 hours' stimulation (nonstimulated, 23.88 ± 7.49 ng/ml of MMP-2 and MIF-stimulated, 51.36 ± 5.55 ng/ml of MMP-2; *P *< 0.05).

To analyze enzymatic activity of RA synovial fibroblast supernatants, a gelatinase assay was performed using fluorescein-labeled gelatin as the substrate (Figure 1b). Fluorescence intensity was determined in cell culture supernatants of RA synovial fibroblasts stimulated with MIF (50 nM) for 24 hours. Gelatinase assay confirmed the enhanced enzymatic activity of MIF-stimulated compared with nonstimulated RA synovial fibroblast supernatants (mean fluorescence, 649 ± 34 versus 503 ± 19, respectively; *P *< 0.05).

Gelatin zymography was performed to visualize the gelatin degradation mediated by MIF in RA synovial fibroblast culture media (Figure 1c). RA synovial fibroblasts were stimulated with TNF-α (1.5 nM) [41] or MIF (50 nM) for 24 hours. Zymography revealed a band of gelatin degradation at 72 kDa, representing pro-MMP-2 protein.

In addition, RA synovial fibroblasts were stimulated with different concentrations of MIF (1 nM, 5 nM, 10 nM, 25 nM, and 50 nM). MMP-2 expression in RA synovial fibroblast supernatants was determined by gelatin zymography. We observed no stimulatory effect at 1 nM MIF, whereas increasing MMP-2 expression was seen in response to higher concentrations of MIF (data not shown). Similarly elevated MMP-2 levels were observed at concentrations of 25 nM and 50 nM MIF.

### MIF-induced MMP-2 production is time-dependent

**Figure 2:**
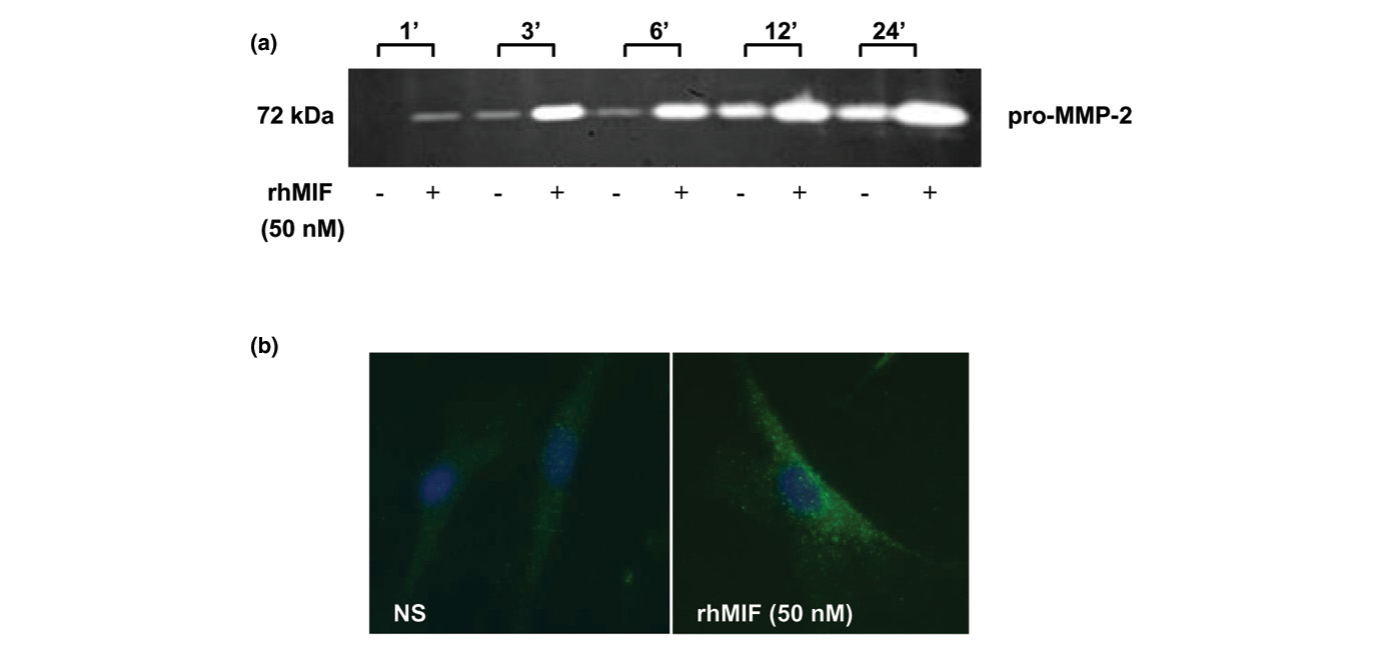
Matrix metalloproteinase (MMP)-2 upregulation by macrophage migration inhibitory factor (MIF) is time-dependent. **(a) **Using gelatin zymography of rheumatoid arthritis (RA) synovial fibroblast culture supernatants, we found MMP-2 upregulation, beginning after 1 hour and increasing continuously over a period of 24 hours. The results represent one of four individual experiments using cells from four donors. **(b) **Immunofluorescence staining of RA synovial fibroblasts for MMP-2 showed a strong perinuclear and discrete diffuse cytoplasmic expression after 1 hour of stimulation by MIF (50 nM; 400×). Results represent one of four individual experiments using cells from four donors. NS, nonstimulated; pro-MMP, pro-matrix metalloproteinase-2; rhMIF, recombinant human macrophage migration inhibitory factor.

We stimulated RA synovial fibroblasts with MIF (50 nM) for different time periods (1 hours, 3 hours, 6 hours, 12 hours, and 24 hours). MMP-2 secretion was visualized in supernatants by zymography (Figure 2a). We found that MIF-induced RA synovial fibroblast MMP-2 upregulation was time-dependent, beginning at 1 hour and increasing continuously over a period of 24 hours. Using immunofluorescence staining, we showed intracellular MMP-2 upregulation after stimulation for 1 hour by MIF (Figure 2b), confirming the role of MIF in MMP-2 induction. Immunofluorescence staining for MMP-2 showed a strong perinuclear and discrete diffuse cytoplasmic pattern.

### RA synovial fibroblast survival

Previously, it has been shown that MIF (5–500 ng/ml) stimulates RA synovial fibroblast proliferation during a 54-hour incubation period [42]. To evaluate whether MIF-mediated MMP-2 production was not simply the result of this effect, cell numbers were evaluated using a CyQuant cell-enumeration kit. Equal RA synovial fibroblast numbers (*n *= 4 donors) were detected in the nonstimulated and MIF-stimulated (50 nM) wells after 24-hours incubation (mean fluorescence intensity, 495 ± 25 versus 478 ± 19, respectively; *P *> 0.05 (data not shown)).

### Decreased production of MMP-2 in *MIF *gene-deficient mice

**Figure 3:**
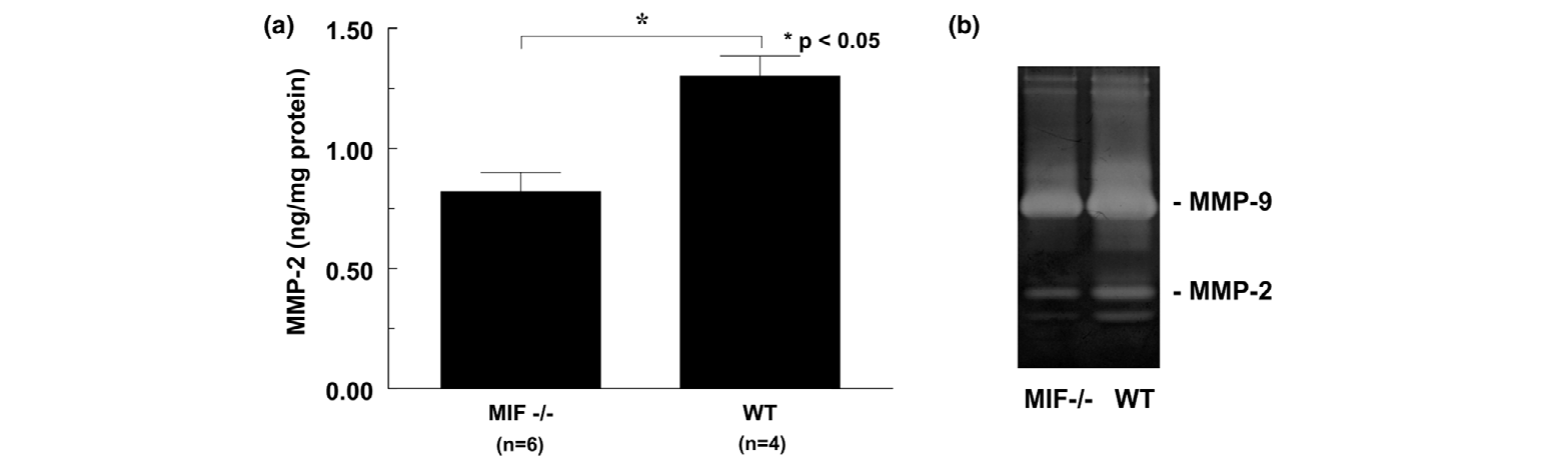
MMP-2 production is decreased in MIF -/- mice compared with WT mice. **(a) **Following zymosan-induced arthritis induction, MMP-2 in mouse knee homogenates was measured by ELISA and normalized to total protein. The mean concentration of MMP-2 ± standard error of the mean (SEM) is represented; *P < 0.05 (*n *= number of mice per group). We found that MIF -/- mice had significantly less joint MMP-2 compared with WT mice. **(b) **Consistent with these results, using gelatin zymography, we found enhanced MMP-2 expression (both the proform and the active form of MMP-2) in antigen-induced arthritis joint homogenates of WT compared with MIF -/- mice. Results are from one representative mouse from each group of four examined. MIF -/-, macrophage migration inhibitory factor gene-deficient; MMP, matrix metalloproteinase; WT, wild-type.

To evaluate the in-vivo role of MIF in MMP-2 production, we induced acute arthritis by intra-articular injection of zymosan in *MIF *gene-deficient and wild-type mice. ZIA is a model of acute inflammatory arthritis with early (day 1) and late (day 14) phases [43]. After 24 hours, mice were euthanized and ZIA knee joints were harvested and homogenized. To compare MMP-2 production in joints of *MIF *gene-deficient and wild-type mice, MMP-2 concentrations of knee homogenates were measured by ELISA and normalized to total protein. We found significantly elevated MMP-2 protein levels in knee homogenates of wild-type mice compared with *MIF *gene-deficient mice (wild-type, 1.3 ± 0.08 ng/mg of protein and MIF gene-deficient, 0.82 ± 0.08 ng/mg of protein; *P *< 0.05), pointing to an important role of MMP-2 in arthritis (Figure 3a).

Additionally, we measured MMP-2 expression in the knee joints of mice after induction of AIA, a different murine model of RA. MMP-2 production of knee homogenates was measured on day 28 after AIA induction using gelatin zymography (Figure 3b). In parallel with our previous results, zymography showed enhanced MMP-2 production in wild-type mice compared with *MIF *gene-deficient mice. Interestingly, zymography revealed both the proform and the active form of MMP-2.

### Immunohistological analysis of MMP-2 expression in ZIA joints

**Figure 4:**
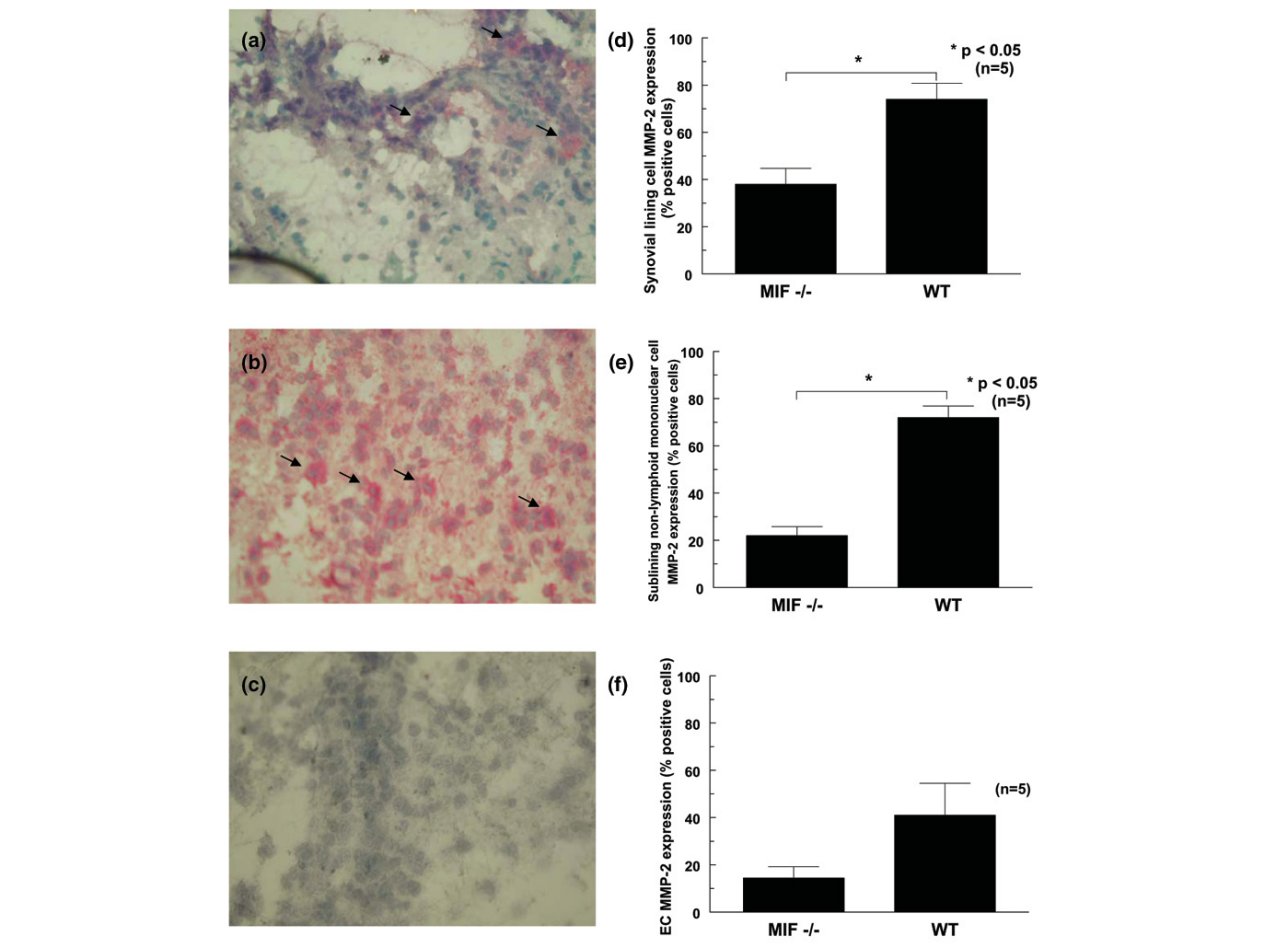
Decreased MMP-2 expression by synovial lining cells in zymosan-induced arthritis (ZIA) joints of MIF -/- mice. **(a–c) **Alkaline phosphatase staining of MMP-2 (red) was performed on frozen ZIA joint sections. MMP-2 expression was decreased in synovial lining cells, which are composed of macrophages and fibroblasts, of MIF -/- **(a) **compared with WT **(b) **mice. Irrelevant immunoglobulin G was used as the negative control **(c) **(400×). Black arrows indicate synovial lining cells stained for MMP-2. **(d–f) **The average percentage of cells stained for MMP-2. The mean percentage of MMP-2 expression ± standard error of the mean (SEM) is shown; *P < 0.05. **(d) **Synovial lining cells showed enhanced MMP-2 expression in WT compared with MIF -/- mice. **(e) **Similarly, MMP-2 was upregulated on sublining nonlymphoid mononuclear cells. **(f) **ECs showed a trend towards MMP-2 upregulation in WT mice ZIA joints, although the difference was not significant (*n *= 5, where *n *= number of animals in each group). EC, endothelial cell; MIF -/-, macrophage migration inhibitory factor gene-deficient; MMP, matrix metalloproteinase; WT, wild-type.

To evaluate the cell-type-specific expression of MMP-2 in the synovium of arthritic joints, we performed immunohistochemistry staining on ZIA joints of *MIF *gene-deficient and wild-type mice. ZIA was induced as we described above, knee joints were kept in OCT (optimal cutting temperature compound), and frozen joint sections were immunoassayed for MMP-2. We found that MMP-2 was mainly expressed by synovial lining cells (Figure 4a–c), sublining nonlymphoid mononuclear cells, and endothelial cells. The immunostaining was quantified as the percentage of cells staining positively for MMP-2. Synovial expression of MMP-2 was enhanced in both lining cells (Figure 4d) and sublining nonlymphoid mononuclear cells (Figure 4e) of wild-type compared with *MIF *gene-deficient mice (synovial lining cells, 74% ± 7 versus 38% ± 7, respectively, and sublining nonlymphoid mononuclear cells, 72% ± 4.9 versus 22% ± 3.8, respectively; *P *< 0.05). A similar trend was seen in endothelial cells, but the difference was not significant (41% ± 13.5 versus 14.4% ± 4.8, respectively; Figure 4f).

### PKCδ, JNK, and Src mediate MIF-induced RA synovial fibroblast MMP-2 production

**Figure 5:**
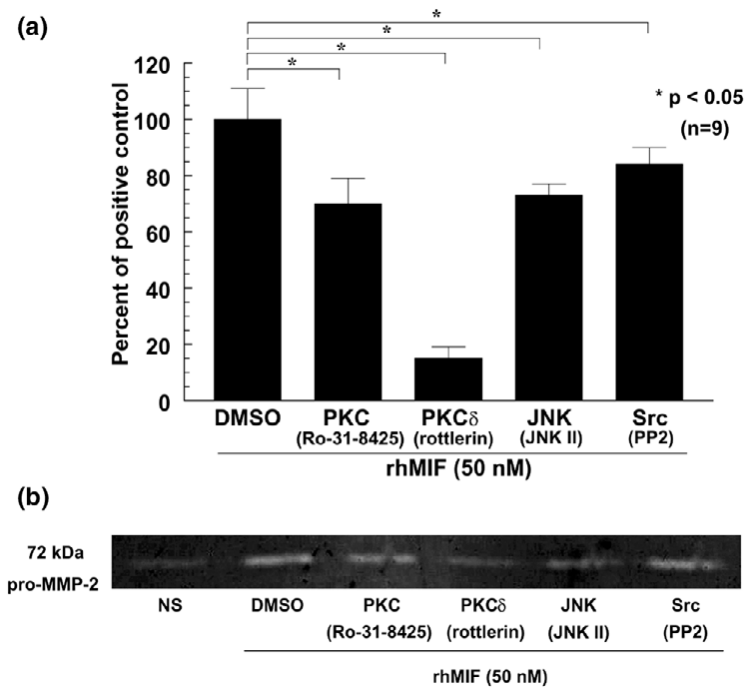
Macrophage migration inhibitory factor (MIF)-induced matrix metalloproteinase (MMP)-2 production is PKCδ, JNK, and Src pathway-dependent. Rheumatoid arthritis (RA) synovial fibroblasts were incubated for 6 hours, with or without MIF (50 nM), in the presence or absence of signaling pathway inhibitors: PKC (pan) inhibitor Ro-31-8425 (1 μM), PKCδ isoform-specific inhibitor rottlerin (1 μM), JNK inhibitor JNK II, and Src inhibitor PP2 (10 μM). **(a) **MMP-2 concentrations in cell culture supernatants were measured by ELISA. Inhibitors to PKCδ, JNK, and Src signaling intermediates inhibited MIF-induced MMP-2 upregulation. **(b) **Gelatin zymography showed the same effect of these inhibitors on MMP-2 upregulation. Results represent three experiments using RA synovial fibroblasts from six donors. DMSO, dimethyl sulfoxide; JNK, c-jun N-terminal kinase; NS, non-stimulated; pro-MMP, pro-matrix metalloproteinase-2; PKC, protein kinase C; rhMIF, recombinant human macrophage migration inhibitory factor.

To examine the signal transduction pathways induced by MIF, RA synovial fibroblasts were stimulated with MIF (50 nM) in the presence of different signaling inhibitors. MMP-2 concentrations in RA synovial fibroblast supernatants were measured by ELISA (Figure 5a) and gelatin degradation was visualized by gelatin zymography (Figure 5b). Several inhibitors were tested, including the PKC inhibitor Ro31-84-25 (1 μM), the protein kinase A (PKA) inhibitor H-8 (10 μM), the MEK inhibitor PD98059 (10 μM), the p38 MAPK inhibitor SB203580 (10 μM), the PI3K inhibitor LY29002 (10 μM), the Src inhibitor PP2 (10 μM), the Jak inhibitor AG 490 (10 μM), the NF-κB inhibitor PDTC (100 μM), the JNK inhibitor SP600125 (10 μM), the G-protein inhibitor pertussis toxin (4.3 nM), and the STAT inhibitor peptide (100 μM). We observed that MMP-2 upregulation by MIF was suppressed by inhibitors of PKC (pan), JNK, and, in part, by Src, suggesting that these signaling pathways are involved in MMP-2 production by RA synovial fibroblasts. To determine the role of different PKC isoforms in MMP-2 production, we used a PKCαβ specific inhibitor, Gö6976 (1 μM), and a PKCδ isoform-specific inhibitor, rottlerin (1 μM). We found that rottlerin inhibited the upregulation of MMP-2 by MIF, whereas the α and β isoform-specific PKC inhibitor Gö6976 did not. By contrast, none of the other specific signaling inhibitors mentioned above reduced MMP-2 expression of MIF-stimulated RA synovial fibroblasts (data not shown).

### MIF induces phosphorylation of PKCδ, JNK, and c-jun in RA synovial fibroblasts

**Figure 6:**
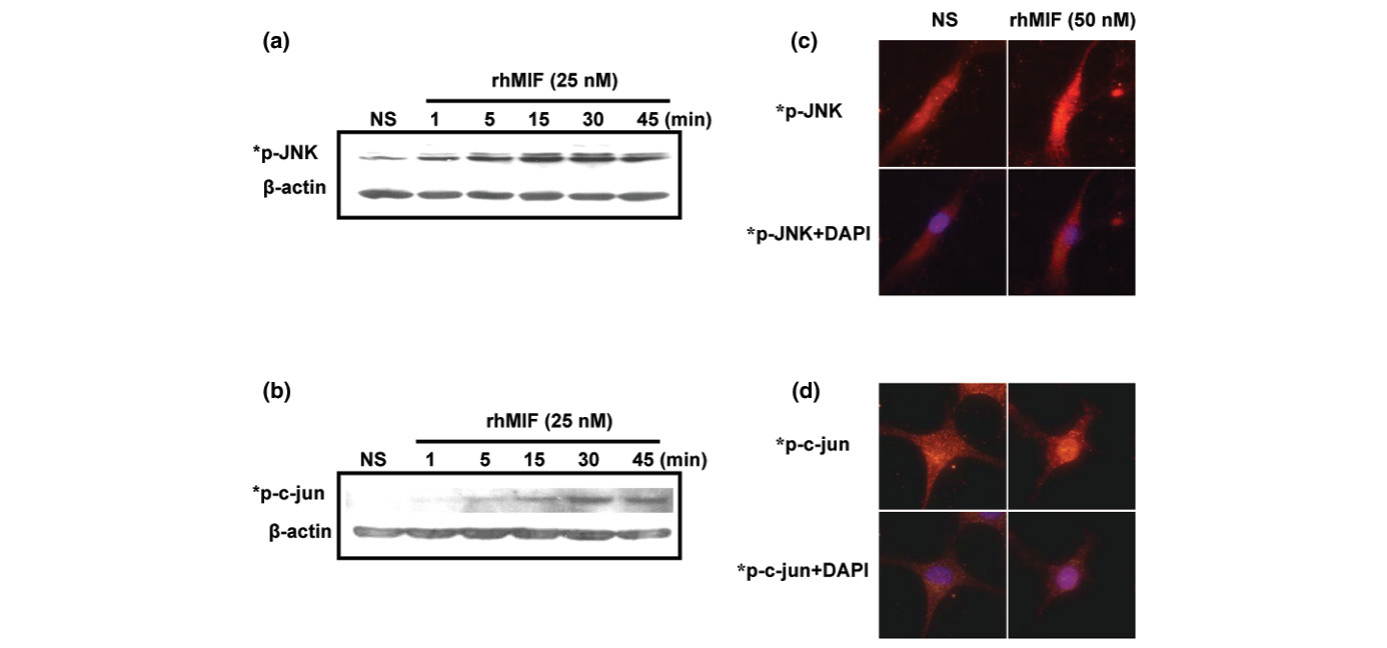
Macrophage migration inhibitory factor (MIF) activates c-jun N-terminal kinase (JNK) and c-jun in rheumatoid arthritis (RA) synovial fibroblasts. **(a–c) **RA synovial fibroblasts were stimulated with MIF (25 nM) for 1 minutes, 5 minutes, 15 minutes, 30 minutes, and 45 minutes. Phosphorylated JNK and c-jun signaling molecule expression in cell lysates was measured by western blot. Blots were stripped and re-probed with antiactin antibody to verify equal loading. MIF-induced phosphorylation of JNK **(a) **and c-jun **(b)**. Results represent one of four similar experiments using cells from four donors. **(c–d) **Immunofluorescence staining of RA synovial fibroblasts shows the expression of different phospho-signaling molecules in the presence or absence of MIF (50 nM) for 25 minutes and with or without DAPI nuclear staining. MIF-induced diffuse cytoplasmic upregulation of *p-JNK **(c)**, increased the nuclear expression of *p-c-jun **(d) **(400×). DAPI, 4',6-diamidino-2-phenylindole dihydrochloride; NS, nonstimulated; *p-JNK, phospho-c-jun N-terminal kinase; rhMIF, recombinant human macrophage migration inhibitory factor; *p-c-jun, phospho-c-jun.

To study signal transduction, we used MIF at a concentration of 25 nM, because we determined this dose to be sufficient for inducing MMP-2 production in RA synovial fibroblasts (see above). RA synovial fibroblasts were stimulated with MIF for different time periods (0 minutes, 1 minute, 5 minutes, 15 minutes, 30 minutes, and 45 minutes; Figure 6). The phosphorylation of JNK and the JNK substrate c-jun was determined by western blot using phospho-specific antibodies. MIF-activated JNK phosphorylation was observed at 1 minute, and a maximum response was seen at 15 minutes (Figure 6a). MIF-induced c-jun activation was observed after 30 minutes (Figure 6b). To confirm this data, we performed immunofluorescence staining of RA synovial fibroblasts using antibodies to phospho-specific signaling molecules. We found diffuse cytoplasmic staining of phospho-JNK in RA synovial fibroblasts stimulated by MIF (Figure 6c). The intracellular localization of phospho-c-jun was primarily nuclear but there was also a small amount of cytoplasmic staining. On MIF stimulation, immunofluorescence staining of phospho-c-jun showed a stronger nuclear pattern, suggesting nuclear translocation of c-jun (Figure 6d).

**Figure 7:**
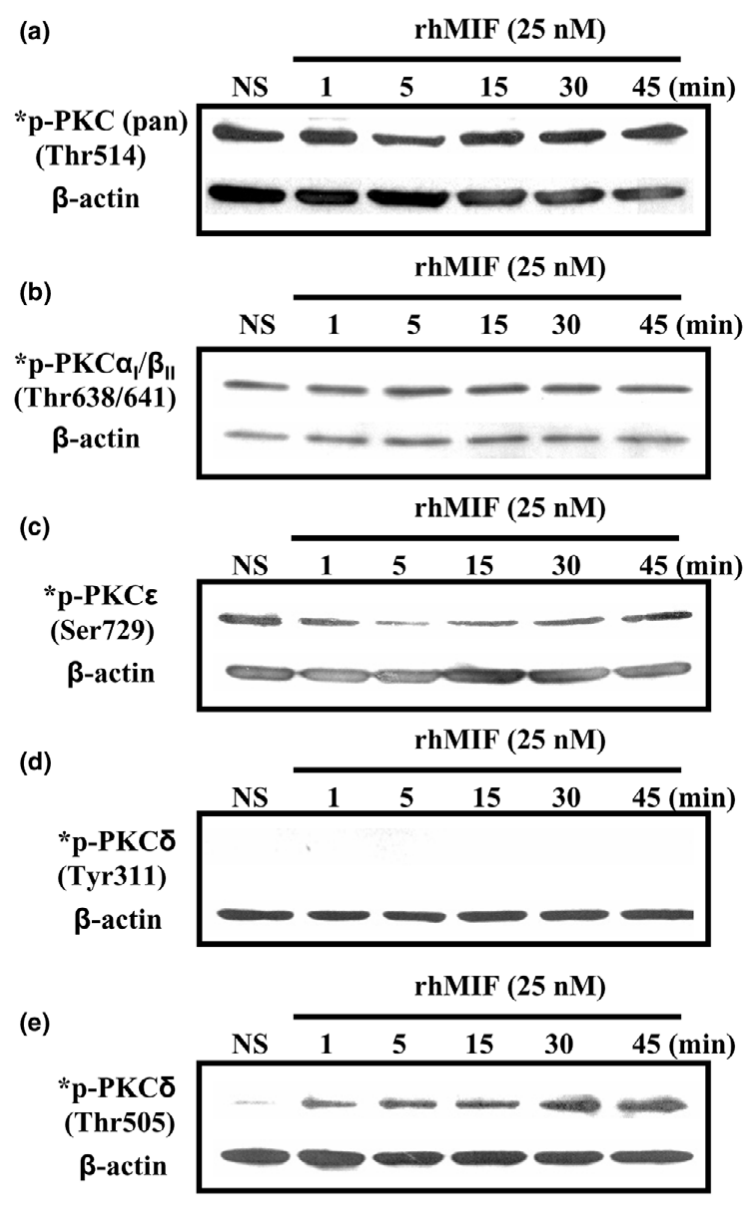
Macrophage migration inhibitory factor (MIF) activates protein kinase C (PKC)δ. Rheumatoid arthritis (RA) synovial fibroblasts were stimulated with MIF (25 nM) for 1 minutes, 5 minutes, 15 minutes, 30 minutes, and 45 minutes. Western blots were performed with phospho-specific antibodies against different isoforms of PKC. *P-PKC (pan) **(a)**, *p-PKCα_I _β_II _**(b) **and *p-PKCε **(c) **were not upregulated by MIF. On the other hand, MIF induced the phosphorylation of PKCδ at Thr505 **(e)**, but not on Tyr311 **(d)**. NS, non-stimulated; p-PKC, phosphor-protein kinase C; rhMIF, recombinant human macrophage migration inhibitory factor.

To determine which PKC isoforms are phosphorylated on MIF stimulation, we used different antiphospho-specific PKC antibodies (Figure 7). We did not find activation of PKC (pan), PKCα_I_β_II_, or PKCε isoforms on MIF stimulation (Figure 7a–c). Also, MIF did not induce phosphorylation of PKCδ at Tyr311 (Figure 7d), but specifically induced phosphorylation of PKCδ at Thr505 (Figure 7e). The reason for this effect could be that PKCδ is not phosphorylated at Tyr311, but at Thr505 instead. MIF-induced activation of this PKC isoform was found after 1 minute, with a maximum response between 30 minutes and 45 minutes.

**Figure 8:**
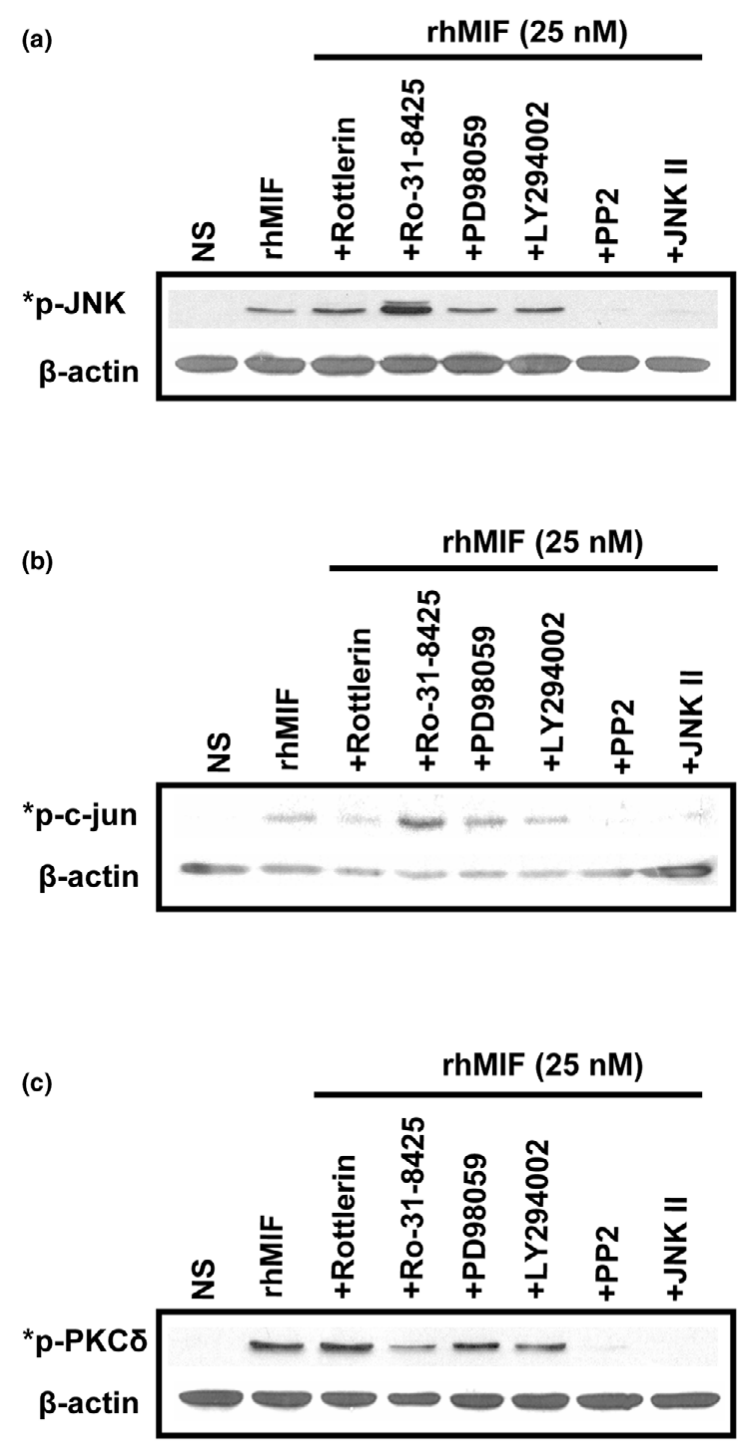
Signaling cascade activated by macrophage migration inhibitory factor (MIF). Rheumatoid arthritis (RA) synovial fibroblasts were pretreated 1 hour before stimulating with MIF (25 nM) for 25 minutes with different signaling inhibitors: the PKCδ inhibitor rottlerin, the pan-PKC inhibitor Ro-31-8425, the MEK (mitogen-activated protein kinase extracellular-signal-regulated kinase) inhibitor PD98059, the phosphatidylinositol 3-kinase (PI3K) inhibitor LY294002, the Src inhibitor PP2, and the JNK inhibitor JNK II. **(a) **Upregulation of *p-JNK by MIF was inhibited by a Src inhibitor PP2. **(b) **The activation of the nuclear factor c-jun required the phosphorylation of JNK and Src. **(c) **Similarly, *p-PKCδ expression was Src and JNK pathway-dependent. Results are representative of four experiments using cells from four donors. JNK, c-jun N-terminal kinase; NS, nonstimulated; p-c-jun, phospho-c-jun; PKC, protein kinase C; rhMIF, recombinant human macrophage migration inhibitory factor.

To determine the downstream and upstream signaling mechanisms, RA synovial fibroblasts were incubated with signaling inhibitors for 1 hour before MIF stimulation (at a concentration of 25 nM). Phosphorylation of JNK was abrogated by the Src inhibitor PP2 (Figure 8a), and c-jun phosphorylation was abrogated by JNK and Src inhibitors (Figure 8b). These data suggest that Src is upstream of JNK, and phosphorylation of JNK leads to the activation of the nuclear protein c-jun. Activation of PKCδ (Thr505) was inhibited by JNK and Src inhibitors (Figure 8c), suggesting that Src and JNK are upstream of PKCδ. The inhibitory activity of rottlerin results from the interaction with the ATP-binding site of PKCδ, which explains why it did not inhibit the phosphorylation of PKCδ [44].

## Discussion

RA is a chronic inflammatory disease characterized by an immunologic disorder that leads to joint destruction. The cellular components of inflamed synovium consist of inflammatory cells, predominantly macrophages, T lymphocytes, and an overgrowth of synovial fibroblasts. RA synovial fibroblasts are key mediators in the pathogenesis of RA because they have the ability to attach to the articular cartilage and invade cartilage [45]. MIF is highly expressed in RA synovium [46], where it regulates proinflammatory cytokines, such as TNF-α, IL-1β, and IFN-γ [25], and induces the production of MMP-1 and MMP-3 in RA synovial fibroblasts [47]. In the present study, a novel role of MIF, the induction of MMP-2 production by RA synovial fibroblasts, and MIF-induced signaling events were analyzed. Previously, we reported the important role of MIF in angiogenesis [30], and the contribution of MIF to arthritis was also shown by independent studies [31,34]. MMPs have the ability to degrade extracellular matrix components, including gelatin, collagens, fibronectin, and laminin [14]. These enzymes have been implicated in several pathologic processes, such as tumor invasion, angiogenesis, atherosclerosis, and RA [3,4,6-10]. In RA, angiogenesis is thought to be a key event in the expansion of the synovial lining of the joints. Angiogenesis requires proteolysis of the extracellular matrix, proliferation, and migration of endothelial cells, in addition to synthesis of new matrix components. MMP-2 has an important role in this angiogenic process [7,19,48]. The evidence for this conclusion is that MMP inhibitors block angiogenic responses both *in vitro *and *in vivo *[49-51].

MIF is thought to be important in the pathogenesis of RA. Previously, we showed the angiogenic potential of MIF both *in vitro *and *in vivo*. MIF induces human dermal microvascular endothelial cell migration and tube formation, and induces angiogenesis in Matrigel plugs (BD Biosciences, San Jose, CA, USA) and in the corneal bioassay *in vivo *[30]. Two groups observed independently that in *MIF *gene-deficient mice or wild-type mice treated with neutralizing antibody against MIF the onset of arthritis was delayed and synovial inflammation was decreased [31,34], although the specific role of MIF in tissue destruction is not clear yet.

In this study, we investigated the effect of MIF on RA synovial fibroblast MMP production. In terms of MMPs and MIF, Onodera and coworkers showed a stimulatory effect of MIF on MMP-1 and MMP-3 mRNA levels in RA synovial fibroblasts [47], and also on MMP-9 and MMP-13 production in rat osteoblasts [52]. Despite Onodera and coworkers finding increased levels of MMP-1 protein in supernatants of MIF-stimulated early passage (passage 3) RA synovial fibroblasts [47], we found no upregulation of MMP-1, MMP-3, and MMP-13 by MIF in later passage (passage 8) cells. It is possible that these differences in responsiveness might result from differences in cell passage number, because similar in-vitro aging effects were shown previously [53].

Cell invasion and angiogenesis are crucial processes underlying diseases such as RA and cancer. Previously, Meyer-Siegler and coworkers showed a positive correlation between MIF and MMP-2 in prostate cancer cells. Addition of MIF to proliferating DU-145 prostate cancer cells resulted in a twofold increase in the relative amount of active MMP-2 [54]. In this study, we show that MIF induces MMP-2 production in RA synovial fibroblasts, which could lead to joint destruction in RA. Using in-vitro methods, including gelatin zymography, ELISA, and immunfluorescence staining of RA synovial fibroblasts, we show that MIF induces MMP-2 production by RA synovial fibroblasts. Stimulation of RA synovial fibroblasts with MIF results in a twofold increase in MMP-2 production. In addition, MIF enhances the gelatinase activity of RA synovial fibroblast-secreted proteins. Zymography analysis demonstrated an increase in pro-MMP-2 protein level in RA synovial fibroblasts stimulated by MIF. It is known, that fibroblasts alone routinely release MMP-2 in its proform. However, co-culture of fibroblasts and monocytes results in the activation of pro-MMP-2 [17,55]. Among other factors, neutrophil elastase is also known to augment the conversion of the 72-kDa form of MMP-2 to the 66-kDa form in lung fibroblasts [55,56].

To evaluate the in-vivo role of MIF in MMP-2 production, we induced acute inflammatory arthritis in *MIF *gene-deficient and wild-type mice with zymosan. The synovial inflammation mediated by zymosan is biphasic, with an initial peak at day 1, followed by a continuous decrease, and a secondary increase at day 14, as previously described using isotopic quantification of joint inflammation *in vivo *[43]. Our results confirmed the important role of MIF in MMP-2 production, because *MIF *gene-deficient mice exhibit less joint MMP-2 than wild-type mice. This observation could contribute to a less severe arthritis in *MIF *gene-deficient mice compared with wild-type mice, as described previously [31,34,57]. In parallel with these results, we measured MMP-2 levels in AIA, a murine model of RA, using gelatin zymography. We found that both the proform and active form of MMP-2 are present in the AIA joints and both forms of MMP-2 are upregulated in wild-type compared with *MIF *gene-deficient mice. As with previous monocyte and fibroblast co-culture studies [17,55,56], these findings also suggest that activation of MMP-2 produced by RA synovial fibroblasts requires the presence of other cell types, possibly monocytes or neutrophils.

Immunohistochemical analysis of ZIA joints revealed that MMP-2 is mainly expressed by synovial lining cells, nonlymphoid mononuclear cells, and endothelial cells in the synovium. In addition, we showed that MMP-2 expression by lining cells and nonlymphoid mononuclear cells is upregulated in wild-type compared with *MIF *gene-deficient mice, suggesting an important role of MIF in MMP-2 induction by these cells.

In terms of in-vivo studies, it was previously shown that progressive joint destruction can be prevented by a novel synthetic MMP inhibitor in rat adjuvant-induced arthritis and also collagen-induced arthritis [58,59]. By contrast, in antibody-induced arthritis, arthritis was found to be more severe in *MMP-2 *gene-deficient compared with wild-type mice [60].

We assessed specific signaling pathways mediating MIF-induced MMP-2 production in RA synovial fibroblasts. We found that MIF-induced RA synovial fibroblast MMP-2 production was decreased in the presence of inhibitors of JNK, PKC, and Src signaling pathways. Pretreatment of RA synovial fibroblasts with a PKCδ isoform-specific inhibitor, rottlerin, suppressed MIF-induced MMP-2 upregulation. Interestingly, we also found that MIF induced the phosphorylation of JNK, c-jun, and PKCδ in RA synovial fibroblasts in a time-dependent manner and activation of JNK and PKCδ by MIF required the interaction of Src. JNK and Src are upstream activators of PKCδ and phosphorylation of JNK leads to the activation of c-jun.

A number of molecules are known to be important in MIF-mediated signaling. Tyrosine kinases, PKC, and NF-κB signaling molecules were reported to be activated by MIF, leading to IL-8 and IL-1β upregulation in RA synovial fibroblasts [26]. Onodera and coworkers showed that MIF-induced MMP-1, MMP-3 and IL-1β mRNA upregulation in RA synovial fibroblasts is inhibited by staurosporine (a broad-spectrum inhibitor of protein kinases such as PKC), a tyrosine kinase inhibitor (genistein), a PKC inhibitor (H-7), and a transcription factor AP-1 inhibitor (curcumin) [47]. In another study, MIF increased MMP-9 and MMP-13 mRNA in rat osteoblasts [52]. Genistein and herbimycin A (two tyrosine kinase inhibitors), a selective MAPK kinase inhibitor (PD98059), and curcumin inhibited MIF-induced MMP-13 mRNA upregulation in rat osteoblasts. Consistent with these results, in rat osteoblasts MIF stimulates phosphorylation of tyrosine, autophosphorylation of Src, activation of Ras, activation of extracellular ERK1/2 (a MAPK), but not JNK or p38, and phosphorylation of c-Jun.

Other regulatory mechanisms, for example, transcriptional and post-transcriptional control of mRNA levels of MMP-2 by MIF could also be important and are currently under investigation. The transcription factors Sp1, Sp3, and AP-2 are functionally important in regulating the expression of the MMP-2 gene [61,62]. Previously, both Sp1 and AP-2 transcription factors were implicated in tumor progression [63,64] and angiogenesis [65]. Among these transcription factors, it is also known that c-jun interacts with Sp1 and the expression of Sp1 is decreased by the PKCδ inhibitor rottlerin, suggesting a possible interaction of Sp1 with PKCδ [66].

However, the function of MMP-2 in RA is not yet clear. Several studies showed an important role of MMP-2 in RA: increased levels of MMP-2 were observed in serum and synovial fluid of patients with RA [20], increased MMP-2 production was associated with enhanced RA synovial fibroblast invasion [18], and, additionally, MMP-2 also participated in angiogenesis [7,19,48]. On the other hand, it is also known that gene polymorphisms for MMP-2 can affect susceptibility to development and/or severity of RA, and mutation of the *MMP-2 *gene causes a multicentric osteolysis and arthritis syndrome [67].

## Conclusion

**Figure 9:**
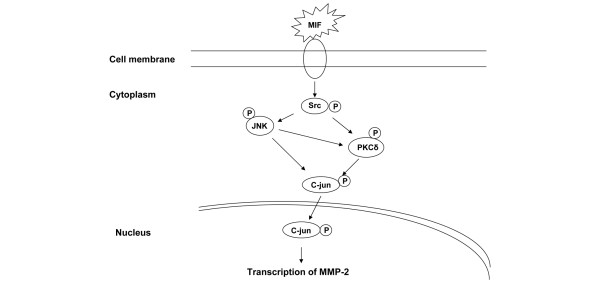
A schematic model of signaling pathways involved in MIF-induced MMP-2 expression in rheumatoid arthritis (RA) synovial fibroblasts. Upregulation of MMP-2 by MIF involves JNK, PKCδ, and Src activation. Activation of JNK and PKCδ by MIF requires Src. Phosphorylation of PKCδ occurs through Src and JNK signaling intermediates. Phosphorylation of PKCδ and JNK leads to the activation and nuclear translocation of c-jun JNK, c-jun N-terminal kinase; MIF, macrophage migration inhibitory factor; MMP, matrix metalloproteinase; P, phosphorylation; PKC, protein kinase C.

To summarize our findings, we demonstrated an important role for MIF in RA synovial fibroblast MMP-2 production, which might contribute to tissue destruction in RA. In-vivo MMP-2 upregulation by MIF was investigated in ZIA, an acute inflammatory arthritis model, and in AIA, a murine model of RA, using *MIF *gene-deficient and wild-type mice. In addition, we describe important pathways activated by MIF leading to MMP-2 upregulation (Figure 9). In our study, we showed that JNK, Src, and PKCδ (a novel signaling intermediate) mediate MIF-induced RA synovial fibroblast MMP-2 expression. Inhibition of MIF and MIF-induced MMP-2 could be potential new therapeutic avenues for RA.

## Abbreviations

AIA = antigen-induced arthritis; BCA = bicinchoninic acid; CO_2 _= carbon dioxide; COX2 = cyclo-oxygenase 2; DAPI = 4',6-diamidino-2-phenylindole dihydrochloride; DMSO = dimethyl sulfoxide; ECL = enhanced chemiluminescence; ED_50 _= Median Effective Dose; ELISA = enzyme-linked immunosorbent assay; FBS = fetal bovine serum; IFN-γ = interferon-γ; IHC = immunohistochemistry; IL-1β = interleukin-1β; Jak = janus kinase; JNK = c-jun N-terminal kinase; MAPK/ERK = mitogen-activated protein kinase extracellular-signal-regulated kinase; mBSA = methylated bovine serum albumin; MIF = macrophage migration inhibitory factor; MMP = matrix metalloproteinase; MT-MMP = membrane-type matrix metalloproteinase; NF-κB = nuclear factor-κB; OCT = optimal cutting temperature compound; PAGE = polyacrylamide gel electrophoresis; PBS = phosphate-buffered saline; PDTC = pyrrolidine dithiocarbamate; PI3K = phosphatidylinositol 3-kinase; PKA = protein kinase A; PKC = protein kinase C; RA = rheumatoid arthritis; SAPK = stress-activated kinase; SEM = standard error of the mean; STAT = signal transducer and activator of transcription; TBST = Tris-buffered saline Tween; TNF-α = tumor necrosis factor-α; ZIA = zymosan-induced arthritis.

## Competing interests

The authors declare that they have no competing interests.

## Authors' contributions

AP designed and carried out most experiments in this study and drafted the manuscript with the assistance of all co-authors. MAA gave critical suggestions concerning experimental design and participated in induction of ZIA. CSH participated in the immunofluorescence staining. RJM participated in cell culture and stimulation of cells. KGH participated in the histopathologic evaluation. EFM and LLS induced AIA and harvested the joints. JRD generated the gene-deficient mice. AEK participated in the design and co-ordination of the study, and is the corresponding author. All authors read and approved the final manuscript.

## References

[B1] Martel-Pelletier J, Welsch DJ, Pelletier JP (2001). Metalloproteases and inhibitors in arthritic diseases. Best Pract Res Clin Rheumatol.

[B2] Krane SM (1995). Is collagenase (matrix metalloproteinase-1) necessary for bone and other connective tissue remodeling?. Clin Orthop Relat Res.

[B3] Herron GS, Werb Z, Dwyer K, Banda MJ (1986). Secretion of metalloproteinases by stimulated capillary endothelial cells. I. Production of procollagenase and prostromelysin exceeds expression of proteolytic activity. J Biol Chem.

[B4] Herron GS, Banda MJ, Clark EJ, Gavrilovic J, Werb Z (1986). Secretion of metalloproteinases by stimulated capillary endothelial cells. II. Expression of collagenase and stromelysin activities is regulated by endogenous inhibitors. J Biol Chem.

[B5] Mannello F, Gazzanelli G (2001). Tissue inhibitors of metalloproteinases and programmed cell death: conundrums, controversies and potential implications. Apoptosis.

[B6] Stetler-Stevenson WG (1999). Matrix metalloproteinases in angiogenesis: a moving target for therapeutic intervention. J Clin Invest.

[B7] Itoh T, Tanioka M, Yoshida H, Yoshioka T, Nishimoto H, Itohara S (1998). Reduced angiogenesis and tumor progression in gelatinase A-deficient mice. Cancer Research.

[B8] Nakagawa T, Kubota T, Kabuto M, Fujimoto N, Okada Y (1996). Secretion of matrix metalloproteinase-2 (72 kD gelatinase/type IV collagenase = gelatinase A) by malignant human glioma cell lines: implications for the growth and cellular invasion of the extracellular matrix. J Neurooncol.

[B9] Cawston TE, Billington C (1996). Metalloproteinases in the rheumatic diseases. J Pathol.

[B10] Cawston TE (1996). Metalloproteinase inhibitors and the prevention of connective tissue breakdown. Pharmacol Ther.

[B11] Jackson C (2002). Matrix metalloproteinases and angiogenesis. Curr Opin Nephrol Hypertens.

[B12] Shamamian P, Schwartz JD, Pocock BJ, Monea S, Whiting D, Marcus SG, Mignatti P (2001). Activation of progelatinase A (MMP-2) by neutrophil elastase, cathepsin G, and proteinase-3: a role for inflammatory cells in tumor invasion and angiogenesis. J Cell Physiol.

[B13] Lafleur MA, Hollenberg MD, Atkinson SJ, Knauper V, Murphy G, Edwards DR (2001). Activation of pro-(matrix metalloproteinase-2) (pro-MMP-2) by thrombin is membrane-type-MMP-dependent in human umbilical vein endothelial cells and generates a distinct 63 kDa active species. Biochem J.

[B14] Curran S, Murray GI (1999). Matrix metalloproteinases in tumour invasion and metastasis. J Pathol.

[B15] Birkedal-Hansen H (1995). Proteolytic remodeling of extracellular matrix. Curr Opin Cell Biol.

[B16] Goldbach-Mansky R, Lee JM, Hoxworth JM, Smith D, Duray P, Schumacher RH, Yarboro CH, Klippel J, Kleiner D, El-Gabalawy HS (2000). Active synovial matrix metalloproteinase-2 is associated with radiographic erosions in patients with early synovitis. Arthritis Res.

[B17] Zhu P, Ding J, Zhou J, Dong WJ, Fan CM, Chen ZN (2005). Expression of CD147 on monocytes/macrophages in rheumatoid arthritis: its potential role in monocyte accumulation and matrix metalloproteinase production. Arthritis Res Ther.

[B18] Zhu P, Lu N, Shi ZG, Zhou J, Wu ZB, Yang Y, Ding J, Chen ZN (2006). CD147 overexpression on synoviocytes in rheumatoid arthritis enhances matrix metalloproteinase production and invasiveness of synoviocytes. Arthritis Res Ther.

[B19] Ohno-Matsui K, Uetama T, Yoshida T, Hayano M, Itoh T, Morita I, Mochizuki M (2003). Reduced retinal angiogenesis in MMP-2-deficient mice. Invest Ophthalmol Vis Sci.

[B20] Yoshihara Y, Nakamura H, Obata K, Yamada H, Hayakawa T, Fujikawa K, Okada Y (2000). Matrix metalloproteinases and tissue inhibitors of metalloproteinases in synovial fluids from patients with rheumatoid arthritis or osteoarthritis. Ann Rheum Dis.

[B21] Petrow PK, Wernicke D, Schulze Westhoff C, Hummel KM, Brauer R, Kriegsmann J, Gromnica-Ihle E, Gay RE, Gay S (2002). Characterisation of the cell type-specificity of collagenase 3 mRNA expression in comparison with membrane type 1 matrix metalloproteinase and gelatinase A in the synovial membrane in rheumatoid arthritis. Ann Rheum Dis.

[B22] Yamanaka H, Makino K, Takizawa M, Nakamura H, Fujimoto N, Moriya H, Nemori R, Sato H, Seiki M, Okada Y (2000). Expression and tissue localization of membrane-types 1, 2, and 3 matrix metalloproteinases in rheumatoid synovium. Lab Invest.

[B23] Bloom BR, Bennett B (1966). Mechanism of a reaction in vitro associated with delayed-type hypersensitivity. Science.

[B24] David JR (1966). Delayed hypersensitivity in vitro: its mediation by cell-free substances formed by lymphoid cell-antigen interaction. Proc Natl Acad Sci USA.

[B25] Calandra T, Bernhagen J, Mitchell RA, Bucala R (1994). The macrophage is an important and previously unrecognized source of macrophage migration inhibitory factor. J Exp Med.

[B26] Onodera S, Nishihira J, Koyama Y, Majima T, Aoki Y, Ichiyama H, Ishibashi T, Minami A (2004). Macrophage migration inhibitory factor up-regulates the expression of interleukin-8 messenger RNA in synovial fibroblasts of rheumatoid arthritis patients: common transcriptional regulatory mechanism between interleukin-8 and interleukin-1beta. Arthritis Rheum.

[B27] Cunha FQ, Weiser WY, David JR, Moss DW, Moncada S, Liew FY (1993). Recombinant migration inhibitory factor induces nitric oxide synthase in murine macrophages. J Immunol.

[B28] Sampey AV, Hall PH, Mitchell RA, Metz CN, Morand EF (2001). Regulation of synoviocyte phospholipase A2 and cyclooxygenase 2 by macrophage migration inhibitory factor. Arthritis Rheum.

[B29] Chesney J, Metz C, Bacher M, Peng T, Meinhardt A, Bucala R (1999). An essential role for macrophage migration inhibitory factor (MIF) in angiogenesis and the growth of a murine lymphoma. Molecular Medicine.

[B30] Amin MA, Volpert OV, Woods JM, Kumar P, Harlow LA, Koch AE (2003). Migration inhibitory factor mediates angiogenesis via mitogen-activated protein kinase and phosphatidylinositol kinase. Circ Res.

[B31] Mikulowska A, Metz CN, Bucala R, Holmdahl R (1997). Macrophage migration inhibitory factor is involved in the pathogenesis of collagen type II-induced arthritis in mice. J Immunol.

[B32] Nishihira J, Koyama Y, Mizue Y (1998). Identification of macrophage migration inhibitory factor (MIF) in human vascular endothelial cells and its induction by lipopolysaccharide. Cytokine.

[B33] Ogawa H, Nishihira J, Sato Y, Kondo M, Takahashi N, Oshima T, Todo S (2000). An antibody for macrophage migration inhibitory factor suppresses tumour growth and inhibits tumour-associated angiogenesis. Cytokine.

[B34] Ichiyama H, Onodera S, Nishihira J, Ishibashi T, Nakayama T, Minami A, Yasuda K, Tohyama H (2004). Inhibition of joint inflammation and destruction induced by anti-type II collagen antibody/lipopolysaccharide (LPS)-induced arthritis in mice due to deletion of macrophage migration inhibitory factor (MIF). Cytokine.

[B35] Koch AE, Polverini PJ, Leibovich SJ (1986). Stimulation of neovascularization by human rheumatoid synovial tissue macrophages. Arthritis Rheum.

[B36] Bozza M, Satoskar AR, Lin G, Lu B, Humbles AA, Gerard C, David JR (1999). Targeted disruption of migration inhibitory factor gene reveals its critical role in sepsis. J Exp Med.

[B37] Keystone EC, Schorlemmer HU, Pope C, Allison AC (1977). Zymosan-induced arthritis: a model of chronic proliferative arthritis following activation of the alternative pathway of complement. Arthritis Rheum.

[B38] Yang YH, Hall P, Milenkovski G, Sharma L, Hutchinson P, Melis E, Carmeliet P, Tipping P, Morand E (2004). Reduction in arthritis severity and modulation of immune function in tissue factor cytoplasmic domain mutant mice. Am J Pathol.

[B39] Laemmli UK (1970). Cleavage of structural proteins during the assembly of the head of bacteriophage T4. Nature.

[B40] Stetler-Stevenson M, Mansoor A, Lim M, Fukushima P, Kehrl J, Marti G, Ptaszynski K, Wang J, Stetler-Stevenson WG (1997). Expression of matrix metalloproteinases and tissue inhibitors of metalloproteinases in reactive and neoplastic lymphoid cells. Blood.

[B41] Nold M, Goede A, Eberhardt W, Pfeilschifter J, Muhl H (2003). IL-18 initiates release of matrix metalloproteinase-9 from peripheral blood mononuclear cells without affecting tissue inhibitor of matrix metalloproteinases-1: suppression by TNF alpha blockage and modulation by IL-10. Naunyn Schmiedebergs Arch Pharmacol.

[B42] Lacey D, Sampey A, Mitchell R, Bucala R, Santos L, Leech M, Morand E (2003). Control of fibroblast-like synoviocyte proliferation by macrophage migration inhibitory factor. Arthritis Rheum.

[B43] Frasnelli ME, Tarussio D, Chobaz-Peclat V, Busso N, So A (2005). TLR2 modulates inflammation in zymosan-induced arthritis in mice. Arthritis Res Ther.

[B44] Gschwendt M, Muller HJ, Kielbassa K, Zang R, Kittstein W, Rincke G, Marks F (1994). Rottlerin, a novel protein kinase inhibitor. Biochem Biophys Res Commun.

[B45] Muller-Ladner U, Kriegsmann J, Franklin BN, Matsumoto S, Geiler T, Gay RE, Gay S (1996). Synovial fibroblasts of patients with rheumatoid arthritis attach to and invade normal human cartilage when engrafted into SCID mice. Am J Pathol.

[B46] Onodera S, Tanji H, Suzuki K, Kaneda K, Mizue Y, Sagawa A, Nishihira J (1999). High expression of macrophage migration inhibitory factor in the synovial tissues of rheumatoid joints. Cytokine.

[B47] Onodera S, Kaneda K, Mizue Y, Koyama Y, Fujinaga M, Nishihira J (2000). Macrophage migration inhibitory factor up-regulates expression of matrix metalloproteinases in synovial fibroblasts of rheumatoid arthritis. J Biol Chem.

[B48] Bendeck MP (2004). Macrophage matrix metalloproteinase-9 regulates angiogenesis in ischemic muscle. Circ Res.

[B49] Murphy AN, Unsworth EJ, Stetler-Stevenson WG (1993). Tissue inhibitor of metalloproteinases-2 inhibits bFGF-induced human microvascular endothelial cell proliferation. J Cell Physiol.

[B50] Anand-Apte B, Pepper MS, Voest E, Montesano R, Olsen B, Murphy G, Apte SS, Zetter B (1997). Inhibition of angiogenesis by tissue inhibitor of metalloproteinase-3. Invest Ophthalmol Vis Sci.

[B51] Benelli R, Adatia R, Ensoli B, Stetler-Stevenson WG, Santi L, Albini A (1994). Inhibition of AIDS-Kaposi's sarcoma cell induced endothelial cell invasion by TIMP-2 and a synthetic peptide from the metalloproteinase propeptide: implications for an anti-angiogenic therapy. Oncol Res.

[B52] Onodera S, Nishihira J, Iwabuchi K, Koyama Y, Yoshida K, Tanaka S, Minami A (2002). Macrophage migration inhibitory factor up-regulates matrix metalloproteinase-9 and -13 in rat osteoblasts. Relevance to intracellular signaling pathways. J Biol Chem.

[B53] Jumblatt MM (1997). PGE2 synthesis and response pathways in cultured corneal endothelial cells: the effects of in vitro aging. Curr Eye Res.

[B54] Meyer-Siegler K (2000). Macrophage migration inhibitory factor increases MMP-2 activity in DU-145 prostate cells. Cytokine.

[B55] Zhu Y, Liu X, Skold CM, Wang H, Kohyama T, Wen FQ, Ertl RF, Rennard SI (2001). Collaborative interactions between neutrophil elastase and metalloproteinases in extracellular matrix degradation in three-dimensional collagen gels. Respir Res.

[B56] Fredriksson K, Liu XD, Lundahl J, Klominek J, Rennard SI, Skold CM (2006). Red blood cells increase secretion of matrix metalloproteinases from human lung fibroblasts in vitro. Am J Physiol Lung Cell Mol Physiol.

[B57] Leech M, Metz C, Santos L, Peng T, Holdsworth SR, Bucala R, Morand EF (1998). Involvement of macrophage migration inhibitory factor in the evolution of rat adjuvant arthritis. Arthritis Rheum.

[B58] Ishikawa T, Nishigaki F, Miyata S, Hirayama Y, Minoura K, Imanishi J, Neya M, Mizutani T, Imamura Y, Naritomi Y (2005). Prevention of progressive joint destruction in collagen-induced arthritis in rats by a novel matrix metalloproteinase inhibitor, FR255031. Br J Pharmacol.

[B59] Ishikawa T, Nishigaki F, Miyata S, Hirayama Y, Minoura K, Imanishi J, Neya M, Mizutani T, Imamura Y, Ohkubo Y (2005). Prevention of progressive joint destruction in adjuvant induced arthritis in rats by a novel matrix metalloproteinase inhibitor, FR217840. Eur J Pharmacol.

[B60] Itoh T, Matsuda H, Tanioka M, Kuwabara K, Itohara S, Suzuki R (2002). The role of matrix metalloproteinase-2 and matrix metalloproteinase-9 in antibody-induced arthritis. J Immunol.

[B61] Qin H, Sun Y, Benveniste EN (1999). The transcription factors Sp1, Sp3, and AP-2 are required for constitutive matrix metalloproteinase-2 gene expression in astroglioma cells. J Biol Chem.

[B62] Wang CH, Chang HC, Hung WC (2006). p16 inhibits matrix metalloproteinase-2 expression via suppression of Sp1-mediated gene transcription. J Cell Physiol.

[B63] Schewe DM, Leupold JH, Boyd DD, Lengyel ER, Wang H, Gruetzner KU, Schildberg FW, Jauch KW, Allgayer H (2003). Tumor-specific transcription factor binding to an activator protein-2/Sp1 element of the urokinase-type plasminogen activator receptor promoter in a first large series of resected gastrointestinal cancers. Clin Cancer Res.

[B64] Schewe DM, Biller T, Maurer G, Asangani IA, Leupold JH, Lengyel ER, Post S, Allgayer H (2005). Combination analysis of activator protein-1 family members, Sp1 and an activator protein-2alpha-related factor binding to different regions of the urokinase receptor gene in resected colorectal cancers. Clin Cancer Res.

[B65] Brenneisen P, Blaudschun R, Gille J, Schneider L, Hinrichs R, Wlaschek M, Eming S, Scharffetter-Kochanek K (2003). Essential role of an activator protein-2 (AP-2)/specificity protein 1 (Sp1) cluster in the UVB-mediated induction of the human vascular endothelial growth factor in HaCaT keratinocytes. Biochem J.

[B66] Chintalgattu V, Katwa LC (2004). Role of protein kinase Cdelta in endothelin-induced type I collagen expression in cardiac myofibroblasts isolated from the site of myocardial infarction. J Pharmacol Exp Ther.

[B67] Martignetti JA, Aqeel AA, Sewairi WA, Boumah CE, Kambouris M, Mayouf SA, Sheth KV, Eid WA, Dowling O, Harris J (2001). Mutation of the matrix metalloproteinase 2 gene (MMP2) causes a multicentric osteolysis and arthritis syndrome. Nat Genet.

